# Necessity of Immediate MRI Imaging in the Acute Care of Severely Injured Patients

**DOI:** 10.3390/medicina57090982

**Published:** 2021-09-18

**Authors:** Daniel Popp, Maximilian Kerschbaum, Daniel Mahr, Claudius Thiedemann, Antonio Ernstberger, Isabel Wiesinger, Wolf Bäumler, Volker Alt, Andreas Schicho

**Affiliations:** 1 Department of Trauma Surgery, University Medical Centre Regensburg, 93053 Regensburg, Germany; maximilian.kerschbaum@ukr.de (M.K.); daniel.mahr@ukr.de (D.M.); claudius.thiedemann@ukr.de (C.T.); volker.alt@ukr.de (V.A.); 2 Department of Trauma Surgery, Clinic Osnabrück, 49076 Osnabrück, Germany; Antonio.ernstberger@ukr.de; 3 Institute of Neuroradiology, Medbo Bezirksklinikum Regensburg, 93053 Regensburg, Germany; isabel.wiesinger@ukr.de; 4 Department of Radiology, University Medical Centre Regensburg, 93053 Regensburg, Germany; wolf.baeumler@ukr.de (W.B.); andreas.schicho@ukr.de (A.S.)

**Keywords:** polytrauma, MRI, acute diagnostic, ISS

## Abstract

*Background and Objectives*: The standard diagnostic procedure for a patient with a suspected polytrauma injury is computed tomography (CT). In individual cases, however, extended acute imaging using magnetic resonance imaging (MRI) can provide valuable and therapy-relevant information. The aim of our cohort study was to find such cases and to describe their characteristics in order to be able to give possible recommendations for MRI application in acute trauma situations. *Materials and Methods*: In the study period from 2015–2019, an evaluation of the imaging performed on polytrauma patients was carried out. The specific diagnostic and therapeutic criteria of the MRI group were further defined. *Results*: In total, 580 patients with an ISS ≥16 (injury severity score) were included in the study. Of these 580 patients, 568 patients received a CT scan and 12 patients an MRI scan as part of the initial diagnostic. Altogether, 66.67% of the MRIs took place outside of regular service hours. The main findings for MRI indications were neurological abnormalities with a focus on myelon injuries. Further MRI examinations were performed to rule out vascular injuries. All in all, 58.3% of the MRIs performed resulted in modified therapeutic strategies afterward. *Conclusions*: MRI in the context of acute diagnostic of a severely injured patient will likely remain reserved for special indications in the future. However, maximum care hospitals with a high flow of severely injured patients should provide 24/7 MR imaging to ensure the best possible care, especially in neurological and blunt vascular injuries.

## 1. Introduction

Acute care of polytraumatized patients and especially the scope of initial diagnosis continues to challenge clinicians around the world. In routine clinical practice, when a potentially life-threatening injury is suspected, a full-body computed tomography (CT) is recommended and performed as a standard of care [1,2,3,4]. This is the most effective and reliable way to quickly assess the extent of the injury and provide detailed information for further therapeutic decisions.

In certain cases, however, an additional MRI is required to provide further information to rule out or confirm the critical diagnosis. For example, MR imaging is used more frequently for expected isolated spinal injuries, also in emergency situations [5,6].

The knowledge gained from this imaging can be crucial for the decision of surgical or conservative treatment. MRI is helpful for patients with post-traumatic spinal cord dysfunction or in case a patient’s symptoms cannot be explained by findings on radiographs or CT or when a reliable neurologic exam cannot be obtained [7,8].

However, MR imaging has several limitations due to its longer scan times, higher expenses, and the need for highly trained staff [7]. Especially the time aspect is considered a limitation in the application for the polytraumatized patient. In addition, there are other mostly unclear factors, such as metal implants (prostheses, pacemakers, etc.). Furthermore, limitations related to the patient’s girth can greatly delay or make MRI imaging impossible. Yet, the guidelines for the care of severely injured patients only provide vague advice on the use of MRI in acute diagnostics, and accordingly, literature addressing the necessity and value of MRI in the acute setting is scarce.

This retrospective cohort study was designed to evaluate the utility of immediate MR imaging in the setting of polytrauma injury care and to define further potential indications and limitations.

## 2. Methods

In this retrospective cohort study, all patients with multiple injuries, who were admitted between 2015 and 2019 to a level one trauma center via shock room of the emergency department, were included. The data were collected by a study assistant on call 24 h/365 d, who was not included in the treatment and diagnostic workup. The study was approved by the institutional ethical review board (14-101-0004).

Inclusion criteria for our evaluation was an ISS ≥16 (injury severity score), graded after complete diagnostics, including computed tomography (CT) and/or MRI.

In order to be able to compare the findings of the MRI group in the context of routine care with an isolated CT, we carried out the evaluations for both CT and MRI.

Patients were divided into 2 groups. Patients who received CT diagnostics exclusively as part of acute diagnostics were assigned to the CT group. Patients who received MRI diagnostics exclusively or additionally were assigned to the MRI group.

Age, gender, ISS, intubation rate, Glasgow Coma Scale (GCS), and the time from admission to the shock room until the end of acute radiologic diagnostic (CT or MRI) were recorded. The demographic data are shown in Table 1. Furthermore, MRI duration and technical extent (No. of sequences acquired; No. of body regions scanned) were tracked. Indication for MRI and additional diagnostic yield compared to clinical and/or CT diagnostics were analyzed. In addition, injury patterns were categorized.

### 2.1. CT-Scan-Protocol

All patients receiving a CT were examined using a 2 × 128 detector row dual-energy scanner (SOMATOM Definition Flash; Siemens Healthcare GmbH, Erlangen, Germany), fulfilling the requirements stated in the national S3—Guideline “Polytrauma/Schwerverletzten—Behandlung”. In our university hospital, this scan is set up as a split-scan, including a dedicated head-and-neck CT-angiogram (10).

First, a non-contrast-enhanced scan of the neurocranium was acquired in 0.75 mm slices (360 mAs, 120 kV, pitch 0.55, increment 0.75 mm, and field of view 230 mm). The second scan was the contrast-enhanced head-and-neck CT-A, triggered in the *A. ascendens*. It reached from the aortic arch covering the outlets of supraaortic branches up to the vertex. Volume and concentration of the contrast agent used (Accupaque 350, GE Healthcare Europe GmbH, Freiburg, Germany) were standardized to a total of 120 mL, with 120 mL injected for the head-and-neck CT-A. The contrast agent injection was followed by a flush with 20 mL of saline. Both contrast agent and saline flush were injected with rates of 3.5 mL/s. ECG-gating is not routinely used in our protocol. The third scan was a venous phase delayed scan, covering the thorax, abdomen, pelvis, and upper half of the thigh; in select cases, the scan length was modified to cover the knees. The scan was performed with a fixed delay of 30 s after the CT-A. The standard field of view was 500 mm; the slice thickness was 5.0 mm; the increment was 5.0 mm; the pitch was 0.60; kV and mAs were calculated and set automatically (CARE kV and CARE Dose; Siemens Healthcare GmbH) based on the scan topogram. A soft-tissue kernel with medium edge attenuation (B26f medium) was used for the calculation of axial, coronal, and sagittal views of the head and neck. Additional thick-slab axial maximal intensity projection (MIP) was rendered (10 mm) axial, sagittal, and coronal. Reconstructions were rendered using a B60f kernel for lung tissue (axial), a B60f sharp kernel for bones (axial and coronal, additional sagittal for spine), and a B30f kernel for soft tissue (axial, additional coronal for abdomen). Further reconstructions were calculated at the radiologists’ discretion depending on the findings or suspicions drawn from the standard datasets.

### 2.2. MRI-Scan-Protocol

MRI scans were performed either on a 1.5 T Symphony or 1.5 T Avanto (Siemens Healthineers, Erlangen, Germany) depending on availability when needed. In accordance with the posed questions, sequences for suspected spinal trauma included at least T1 TSE sagittal, T2 TSE sagittal and axial, T2 STIR sagittal and for suspected cervical artery injury, PD fat-sat axial (neck), DWI/ADC (head) axial, T2 FLAIR (neck and head) axial, contrast-enhanced MR-angiography or time-of-flight (TOF) MR-angiography (neck). Additional sequences were acquired at the discretion of the board-certified radiologist in charge.

Image interpretation (CT and MRI) was performed using a standard three-monitor workstation using the Syngo and Syngo.via picture archiving and communication system (PACS; Siemens Healthcare GmbH, Erlangen, Germany). All imaging studies were read and validated by a board-certified radiologist.

### 2.3. Statistical Analysis

A descriptive statistical analysis was performed. Due to the small cohort size of the MRI group, we were unable to perform meaningful statistical analyses.

## 3. Results

### 3.1. Demographic Data

In total, 580 patients met the inclusion criteria (group CT: 568; group MRI: 12). In the CT group, the majority of patients were male (72.2%). The MRI group showed an equal gender distribution (Table 1). Ten patients in the MRI group received CT and MRI-scan, two patients received MRI only. The average age in the CT group was 48.96 years ± 21.82 and 45.45 years ± 22.22 in the MRI group. The average ISS was almost identical in both groups with 30.60 ± 15.86 and 32.73 ± 17.92, respectively. The GCS also showed no significant difference when comparing the two groups (10.71 ± 4.84 vs. 11.75 ± 4.40). Patients in the CT group showed a significantly higher preclinical intubation rate compared to the MRI group (60.3% vs. 25.0%).

### 3.2. Timing of MRI

Table 2 shows the maintenance of the MRI times. A distinction was made between the day shift, night shift, and weekend shifts. It can be seen here that the majority of MRI examinations were carried out outside of regular working hours (66.67%). Less than half of the examinations were performed during the day shift.

### 3.3. Injury Overview

Table 3 presents the distribution of injuries of the study population, comparing the CT- and MRI-group. A significant difference in the incidence of spinal and cervical injuries was observed (spine injury 16.9% vs. 83.3%, neck injury 4.2% vs. 25%). There were no differences in injury incidence in the head, face, thorax, abdomen, extremities, pelvis, or soft tissue injuries.

Table 4 shows a detailed breakdown of the MRI examinations performed. There were six women and six men in the group. The youngest patient was 17 years old; the oldest patient was 80 years old at the time of the accident. The mean age was 44.67 ± 21.36 years. The average time from MRI start to end was 31.83 ± 21.29 min. In seven out of twelve cases, the evaluation of the MRI resulted in consequences for further treatment, which differed from the initial CT diagnosis.

**Figure 1:**
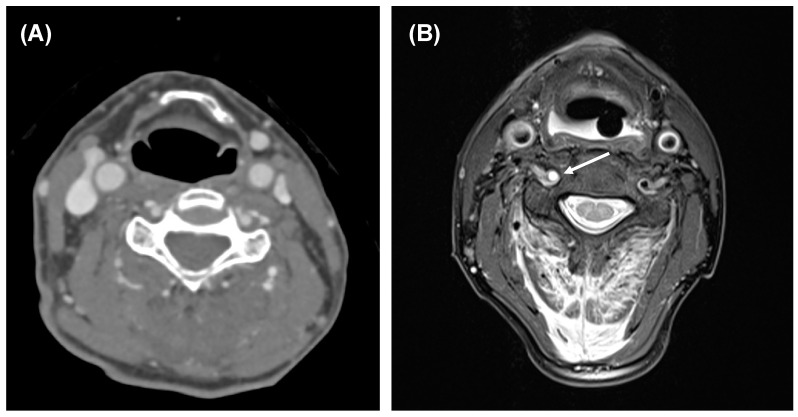
CT and MRI of a 67-year-old male after a car accident. (**A**) CT including CT-A revealed a fracture of C2. (**B**) Additional MRI showed a dissection of the right vertebral artery (arrow; proton density image with fat-saturation), progressed in the meantime from a smaller injury not detectable in CT-A. Furthermore, MRI showed additional fractures of C4 and C5 and a myelon damage level C2.

Figure 1 shows an example case. This shows the information gain of the MRI compared to an initially inconspicuous CT.

## 4. Discussion

MRI imaging as part of the acute diagnosis of a polytrauma patient is rarely indicated in routine clinical practice. In this study, we were able to evaluate special indications for this imaging.

Overall, it should be noted that there is currently very little meaningful literature on MRI diagnosis in polytrauma patients. Thus, our study can provide a valuable contribution to the development of guidelines in acute diagnostics.

By far, the leading indication for MRI in our patients in the acute diagnosis of polytrauma was spinal injury with concomitant neurologic symptoms (66.67%). This is consistent with the recommendations of Sarani et al., O’Connor et al., and Chandra et al., who recommended adding further MR imaging in patients with unremarkable CT of the spine but abnormal neurology [5,7,9]. However, only isolated spinal injuries were considered here and not polytraumatized patients as in our work. Khurana et al. demonstrated that in CT-graphically proven fractures in the region of the thoracic and lumbar spine, 15% of other acute trauma sequelae could be visualized on MRI, whereby important information for planning the following therapy could be obtained [9]. In this study, too, only isolated spinal injuries were examined.

In our study, it is noticeable that the patients in the MRI group showed a lower prehospital intubation rate. In our view, one possible explanation is that, especially in the case of unclear neurological abnormalities, which turned out to be one of the main indications for MRI in our study, the patient was transported awake for better clinical assessment. Another explanation might be due to the somewhat more difficult handling of patients requiring ventilation in MRI. A lower ISS was, therefore, not a reason to perform an additional MRI.

Though, to date, studies on MRI examinations of the spine in the setting of polytrauma do not exist. With our results, we can provide initial insights into this. The second most common indication was advanced diagnostic of cervical vascular injuries (25%). Figure 1 shows a clinical application with therapy-relevant information gained through MRI. In a previous paper, we were able to demonstrate the benefit of this imaging in the context of cervical vascular diagnostics and recommended the implementation of this imaging option [10] in clinical routine. This approach is reinforced by Yamada et al., who proposed a concept of early MRI diagnostic in suspected lesions of cervical vessels, allowing an earlier administration of treatment, which, in turn, contributes to a good clinical outcome [11]. 

An important point in the context of polytrauma diagnostics is the time factor. Here, there was a clear difference between the CT and MRI group. The door-to-image time of the CT group was 27.48 ± 8.03 min compared to 111.45 ± 45.9 min in the MRI group. This is due to the fact that ten patients first underwent a CT and only then an MRI. Patients who are in a cardio-pulmonary unstable state due to the injury can, therefore, not receive MRI diagnostics as part of acute diagnostics. Depending on the CT findings, these patients must be transferred quickly to the operating theater for initial treatment or into the hands of the intensive care specialists. After stabilization, MRI imaging can then be performed for diagnostic expansion if required.

In our opinion, the main arguments against the use of MRI, including whole-body MRI, in the shock room phase are availability and the possibly considerable delay in therapy due to excessively long examination times. MRI has a high value after the secondary survey or in the follow-up because of the comparable or higher sensitivity in intracranial or spinal injuries [12]. An emergency MRI has already been increasingly implemented clinically during core working hours as part of acute neurological diagnostics. Wenger et al. described specific emergency sequences, which should be used in acute situations to extend the diagnostics [13]. Acute diagnostics should also be carried out in the trauma scenario in close cooperation with a radiologist in order to incorporate any necessary additional sequences into the MRI diagnostics, as already described by Wenger et al. [13]. In our view, it also makes sense to have such emergency sequences available for trauma situations so that no time is lost when needed.

For regional and local trauma centers, 24/7 MRI availability presents a difficult problem to solve. Due to the infrequent indication from the shock room, even with high polytrauma numbers, 24/7 availability should initially be sufficient at level one trauma centers. Fixed and routine implementation of MRI imaging in the polytrauma algorithm is necessary due to the heterogeneous volume of injuries and the often associated acute need for surgery. As for the trauma-CT-scans, different imaging protocols can be established. For MRI, the variety of options presents a significant challenge. We, for now, recommend sagittal T1, T2, and T2 STIR plus axial T2 in suspected spine trauma. For cervical artery dissections, axial PD and T1 fat-sat plus contrast-enhanced MR-angiography and/or time-of-flight MR-angiography, especially for dissections near or within the skull base, revealed a lot of dissections in a step-up approach [10]. Fast whole-body sequences, e.g., DWIBS (diffusion-weighted imaging with background suppression), should be evaluated as an add-on for patients undergoing spine MRI anyways or for child trauma MRI. The latter remains a point of debate with little evidence and no recommendations for when and how to use it as it is very time-consuming and logistically challenging.

Two further indications for MRI diagnostics, which were not observed in our study, is the implementation in pediatric diagnostic. Hauwe et al. highlighted the relevance of MRI imaging and equivalent radiation reduction in pediatric diagnosis [8]. The DGU Spine Trauma Working Group recommended MRI imaging in the cardio-pulmonary stable child and suspected monotrauma of the spine [14]. In a study by Ferrazzano, the use of MRI imaging in children with severe intracranial hemorrhage was reported to be high in percentage. However, it was mostly performed in the centers within the first 7 days after trauma and not directly as part of the acute diagnostic workup. He also mentioned that there are currently no guidelines for the diagnostic use of MRI in severely injured pediatric patients [15]. Gordic et al. were able to describe a positive benefit of MRI diagnostics in the diagnosis of traumatic splenic lacerations [16].

The main limitation of our study is the large difference in group size, which means that limitations in statistical significance must be taken into account.

Another limitation of our study is that no compelling indications for extended acute MRI imaging can be given in the context of polytrauma care. In our view, this is explained by the great heterogeneity of the injury patterns of these patients. For example, a patient may show symptoms of an incomplete transverse section, which cannot be assigned to an exact level and thus require further imaging, but a simultaneously existing splenic rupture with cardio/pulmonary instability requires immediate surgical treatment. However, we were able to show typical red flag injuries (spinal injury and cervical artery dissections), which should be diagnosed by MRI in cardio/pulmonary stable patients.

Already in 2009, Provenzale et al. concluded in their review that sensitivity and specificity of MR-angiography and CT angiography are similar for the detection of cerebrovascular injuries in blunt trauma [17]. With recent technical improvements for CT-A protocols and scanners, detection rates of cervical artery dissections rise [18]. In parallel, MR imaging is speeding up significantly and can offer additional or confirmatory information in doubtful cases [10,19]. To our knowledge, a prospective study comparing state-of-the-art MR-A and CT-A is missing.

## 5. Conclusions

MRI in the context of the acute diagnostic of a severely injured patient will likely remain reserved for special indications in the future. In this special patient clientele, the clinical condition of the patient is the decisive limiting factor for time-consuming MRI diagnostics. However, maximum care hospitals with a high flow of severely injured patients should provide 24/7 MR imaging to ensure the best possible care, especially in neurological and blunt vascular injuries.

## Figures and Tables

**Table 1:** Demographic data of analyzed patients. ISS—injury severity score, GCS—Glasgow Coma Scale, SD—standard deviation.

	CT Group	MRI Group
Number (*n*)	568	12
Male (*n*/%)	410/72.2%	6/50.0%
Age (years ± SD)	48.96 ± 21.82	44.67 ± 21.36
ISS (∅ ± SD)	30.60 ± 15.86	32.73 ± 17.92
GCS (∅ ± SD)	10.71 ± 4.84	11.75 ± 4.40
Intubation (*n*/%)	317/60.3%	3/25.0%
Door-to-image (min ± SD)	27.48 ± 8.03	111.45 ± 45.9

**Table 2:** Breakdown of when the MRI was performed. Differentiation between dayshift, nightshift, and weekend. In total, 81.8% of patients in the MRI group received a CT in the initial diagnostic phase. Two patients received solely MRI diagnostics.

Working Hours	Number	Percent (%)
Dayshift Monday-Friday; 8 a.m.–4 p.m.	4	33.33
Nightshift Monday-Friday; 4 p.m.–8 a.m.	6	50
Weekend Saturday + Sunday	2	16.67

**Table 3:** Injuries in the respective body area with AIS ≥ 3 in the course of polytrauma injury with ISS ≥ 16.

	CT Group	MRI Group
Head (*n*/%)	286/50.4	5/41.7
Face (*n*/%)	62/10.9	0/0
Thorax (*n*/%)	277/48.8	5/41.7
Abdomen (*n*/%)	79/13.9	0/0
Spine (*n*/%)	96/16.9	10/83.3
Upper extremities (*n*/%)	28/4.9	0/0
Lower extremities (*n*/%)	117/20.6	0/0
Pelvic (*n*/%)	87/15.3	2/16.7
Neck (*n*/%)	24/4.2	3/25
Soft tissues *(n*/%)	6/1.1	0/0

**Table 4:** Evaluation of MRI-specific data.

Sex	Age in Years	Diagnosis MRI (Non Detectable by CT)	Therapeutic Consequence	Duration in Min	No. of Sequences
m	36	Lig. flavum rupture, epidural hematoma, Lig. long. post. Rupture, fracture T11	y	16	3
m	19	None	n	42	10
m	40	None	n	24	6
f	17	Myelopathy, vertebral artery injury	y	43	6
m	18	Internal carotid artery injury	y	20	5
f	50	Myelopathy, Lig. Long. Ant. and post. Rupture	y	80	7
f	75	Discal injury, myelopathy	y	15	4
m	47	Myelopathy	n	51	7
f	80	Myelopathy	n	11	4
f	50	Internal carotid artery injury bilateral, stroke	y	16	8
f	37	Epidural hematoma, myelopathy	n	14	5
m	67	Fracture T4+5, vertebral artery dissections, myelopathy	y	50	11

## Data Availability

The data are not publicly available due to Data protection which is required by the Ethics Committee.
